# Optimal Cooperation-Trap Strategies for the Iterated Rock-Paper-Scissors Game

**DOI:** 10.1371/journal.pone.0111278

**Published:** 2014-10-29

**Authors:** Zedong Bi, Hai-Jun Zhou

**Affiliations:** State Key Laboratory of Theoretical Physics, Institute of Theoretical Physics, Chinese Academy of Sciences, Beijing, China; Wenzhou University, China

## Abstract

In an iterated non-cooperative game, if all the players act to maximize their individual accumulated payoff, the system as a whole usually converges to a Nash equilibrium that poorly benefits any player. Here we show that such an undesirable destiny is avoidable in an iterated Rock-Paper-Scissors (RPS) game involving two rational players, X and Y. Player X has the option of proactively adopting a cooperation-trap strategy, which enforces complete cooperation from the rational player Y and leads to a highly beneficial and maximally fair situation to both players. That maximal degree of cooperation is achievable in such a competitive system with cyclic dominance of actions may stimulate further theoretical and empirical studies on how to resolve conflicts and enhance cooperation in human societies.

## Introduction

The solution concept of Nash equilibrium (NE) plays a fundamental role both in classic game theory and in evolutionary game theory [1]–[4]. This concept is developed under the assumption that the players of a game system are sufficiently rational, so that they are able to learn accurately the strategies of the competing players and to optimize their own strategy accordingly. A Nash equilibrium is then a point in the strategy space of the game system such that any single player is unable to achieve better performance by changing her/his own strategy in any arbitrary way.

Many non-cooperative games have only a unique NE. When such a game is played by highly rational players who act to maximize their individual accumulated payoff, it is unavoidable that the system will sooner or later converge to this unique equilibrium situation. Unfortunately, however, it is usually the case that the NE of a non-cooperative game is an unfavorable or even miserable destiny for all the players. Let's consider the two-player Prisoner's Dilemma (PD) game as a simple example. The cooperative situation of both players choosing not to confess is much better than the defection situation of both players choosing to confess, but the latter is the unique NE of this game while the former is not [5]. The Nash equilibrium theory therefore predicts that cooperation is unlikely to sustain when rational players face the conflict between self-interest and group benefit. Yet cooperation is actually a ubiquitous phenomenon of human society at all levels, and it is also widely observed in various biological systems. Researchers have been puzzled by these facts very much for many years, and they have proposed a long list of microscopic mechanisms trying to explain the promotion and maintenance of cooperation [6]–[8].

**Figure 1:**
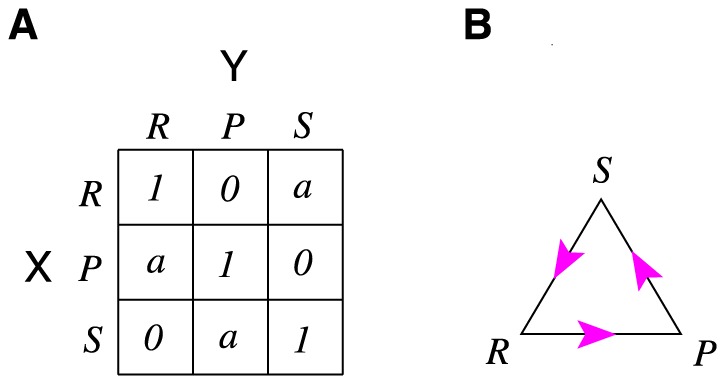
The Rock-Paper-Scissors game. (A) The payoff matrix. Each matrix element is the payoff of the row player X's action in competition with the column player Y's action. (B) The cyclic (non-transitive) dominance relationship among the three candidate actions: Rock (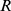) beats Scissors (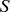), 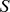 beats Paper (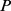), and 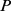 in turn beats 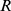.

In this paper we study the issue of cooperation in the itererated two-player Rock-Paper-Scissors (RPS) game, which is a fundamental non-cooperative game with cyclic dominance among its action choices (namely Rock beats Scissors, Scissors beats Paper, and Paper in turn beats Rock), see Fig. 1. This game has been widely used to study competition phenomena in society and biology, especially species diversity and pattern formation (see, for example, references [9]–[12]). While the NE theoretical framework assumes that the rational players of such a game behave *passively* in the sense that they try to maximize individual gains by making best responses to the inferred/experienced strategy of the opponent, we assume that one of the players might act more *proactively*. An intelligent and rational player may ask the following question: how should I design my own strategy so that my rational opponent(s), in best response to me, for sure will adopt certain strategy that is most beneficial to me? In later discussions we refer to such a strategy as a cooperation-trap (CT) strategy, as it has the effect of trapping an opponent in a cooperation state. When optimized, such a CT strategy offers high and maximally fair accumulated payoffs to both players.

In a literature search for related studies, we found that an early paper of Grofman and Pool [13] investigated cooperation in the PD game from the same angle of intelligent design. In this pioneer but largely forgotten paper, the authors proved that a partial Tit-for-Tat strategy [14] has the potential of enforcing cooperation in the two-player iterated PD game. The Win-stay, Lose-shift strategy [15], [16] can also be analyzed in a similar way.

The present effort can be regarded as an extension of the Grofman-Pool theory to the iterated RPS game, which has the additional difficulty of having more than two action choices that are related by a rotation symmetry (see Fig. 1B). This same theoretical framework may also be applicable to many other two-player iterated non-cooperative games, and it may serve as a guiding principle of designing fair solutions or strategies for the purpose of resolving conflicts and enhancing cooperation in human societies.

## Results

### The Rock-Paper-Scissors game

Consider two players X and Y playing the RPS game for an indefinite number of rounds. At every game round each player can choose one action among three candidate actions 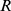 (rock), 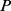 (paper) and 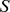 (scissors). This game has only a single parameter, the payoff 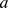 (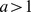) of the winning action (see Fig. 1A). For example, if the player X chooses action 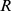 in one game round and her opponent Y chooses action 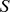, then X wins with payoff 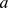 and Y loses and gets zero payoff; if the competition is a tie with both players choosing the same action, each player gets unit payoff.

When 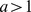 the system has only a unique NE and it is mixed-strategy in nature, namely each player chooses the three actions with equal probability 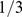 at every game round independently of each other and of the prior action choices [2]. In this mixed-strategy NE the expected payoff per round (EPR) for each player is then simply 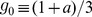. We refer to 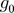 as the NE payoff. For 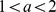 the NE payoff is less than the unit payoff value each player would get if both players choose the same action in every game round, and consequently the NE is evolutionarily unstable in this parameter range. When 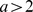 the NE mixed strategy outperforms the pure strategy of both players choosing the same action, and the NE is then evolutionarily stable [3], [4], [17] and it is the converging point of various dynamical learning processes [18].

### Memoryless cooperation-trap strategies

We now develop CT strategies for player X, and begin with the simplest case of memoryless strategies, namely at every game round player X does not consider her and her opponent's prior actions nor the outcomes of prior plays but chooses actions 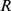, 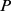 and 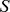 according to the corresponding probabilities 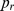, 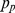, and 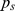 (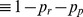), which are fixed by player X at the beginning of the whole game. Without loss of generality we assume that 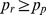 and 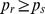, i.e., action 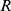 is a favoriate choice of X.

As player Y is sufficiently intelligent, he will figure out the strategy of X after a small number of game repeats. Alternatively, with the aim of promoting cooperation from player Y, player X may also explicitly inform Y about her strategy parameters, which are 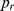 and 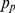 in the present case. And since Y is sufficiently rational, he then for sure will adopt the optimized probabilities 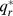, 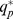, and 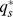 (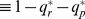) of choosing the three actions 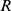, 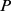 and 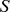. The EPR 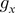 of player X and the optimized EPR 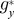 of player Y are 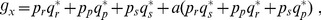
(1)

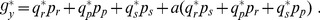
(2)


If the strategy of player X have the following property that 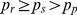, then because action 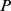 is strictly the least favored choice of player X, then player Y realizes that it is of his best interest to choose action 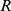 in every game round (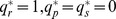) if 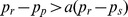 but to choose action 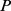 in every game round (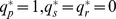) if 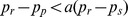. In other words, player X traps player Y to stay in a pure strategy which has maximal degree of predictability. Player X of course should choose the strategy parameters 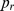 and 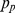 to maximize her EPR 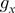 under the constraint of not destroying the nice trapping effect of her strategy. It is not difficult to verify the following conclusions:

1. If the payoff parameter 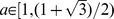, the optimal CT strategy is 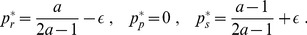
(3)(Here and in latter discussions, 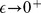 is an arbitrarily small positive value.) The associated maximal EPR for player X is 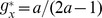, while player Y is very satisfied with sticking to action 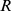 and getting a larger EPR of 
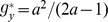
. To give a concrete example, at 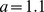 we have 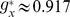 and 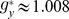, which are considerably larger than the NE payoff 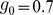.
2. If 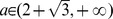, the optimal CT strategy is 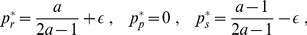
(4)The associated optimal EPR of player X is 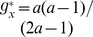, while player Y receives a larger EPR value of 
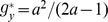
 by sticking to action 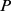. Notice that when 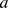 is sufficiently large, 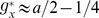 and 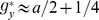, which are almost 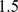 times that of the NE payoff 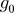.

**Figure 2:**
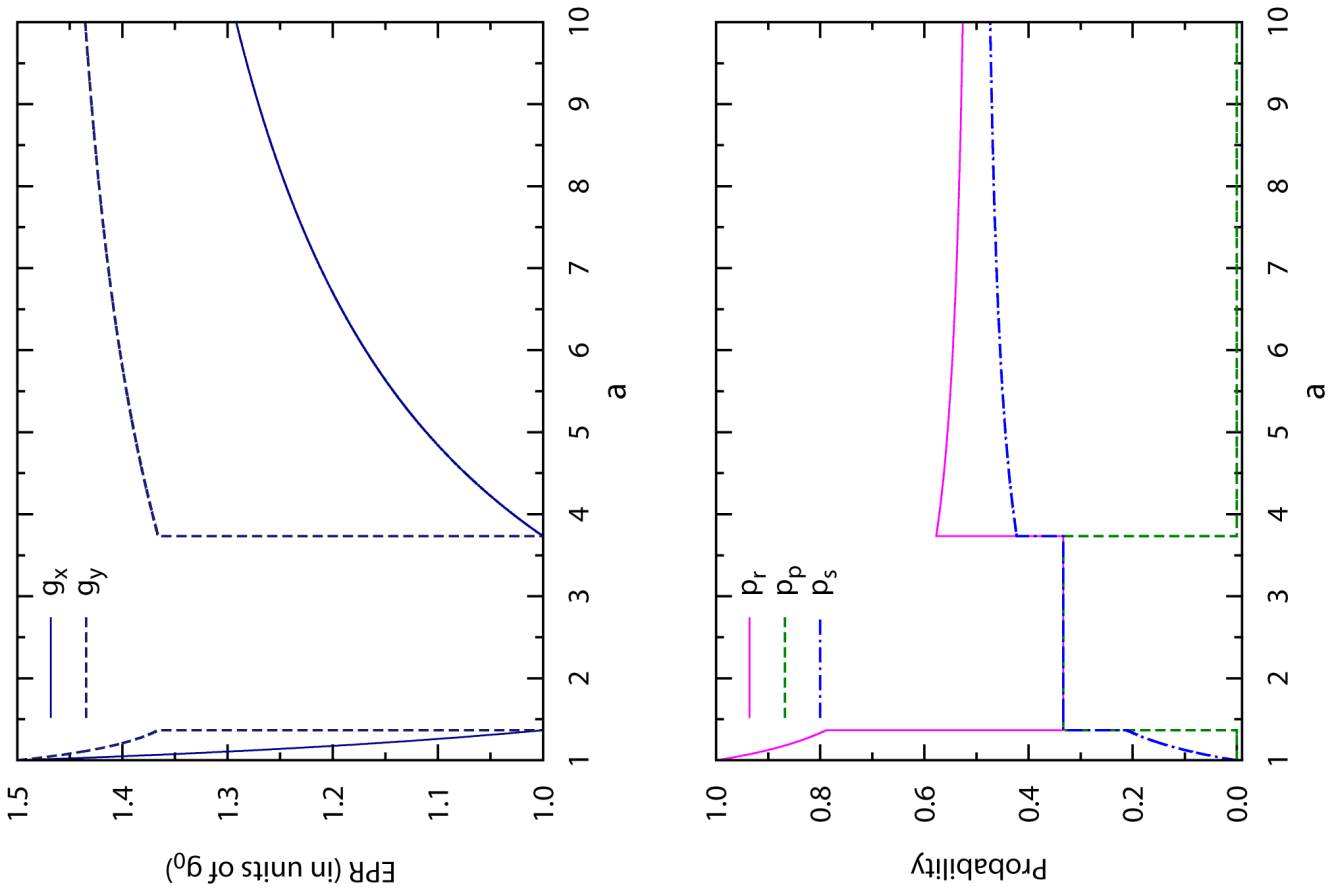
Optimal memoryless CT strategy. The optimal values of both players' expected payoff per round 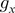 and 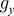 are shown in the upper panel (in units of NE payoff 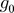) for each fixed value of 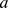, while the optimal values of the CT strategy's choice probabilities 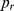, 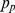 and 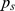 are shown in the lower panel. When 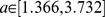 the NE mixed strategy is better for player X than the CT strategy.

Figure 2 gives a direct view about how the optimal EPRs of both players and the optimal CT strategy of player X change with 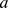. This optimal memoryless CT strategy indeed offers both players higher accumulated payoffs than the NE mixed strategy does. However, the passive player Y benefits more than the proactive player X. It is then natural for player X to feel that she has sacrificed too much for enforcing cooperation and to declare that such a CT strategy, although better than the NE mixed strategy, is unfair as her opponent earns more by free riding. Furthermore, this CT strategy is worse than the NE mixed strategy in the parameter range of 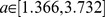.

These shortcomings of the memoryless CT strategy can be eliminated by increasing the memory length of the CT strategy.

### Cooperation-trap strategies with finite memory length

Recent laboratory experiments carried at Zhejiang University [19] revealed that decision-making of human subjects has strong memory effect, namely the payoffs of the previous game rounds influence considerably a player's action choices in the following game rounds. For the RPS game, the implications of such conditional response strategies have not yet been fully explored. Here we suggest that the proactive player X can adopt an optimized version of such a strategy to enforce fair cooperation.

When the payoff parameter 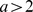, a play output of win-lose brings payoff 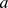 to the group, while a tie output only brings lower payoff 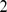. Therefore it is desirable for player X to discourage the occurrence of tie output. For the simplest case of unit memory length, the CT strategy then goes as follows: If player X wins over or loses to player Y in the previous game round, then in the next round she chooses action 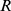 with probability 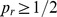 and action 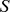 with probability 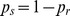 (she avoid choosing action 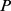, i.e., 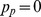); but if X ties with Y in the previous game round, then in the next round she chooses the three candidate actions with equal probability 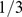. This strategy has only a single parameter 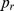. The motivation for player X to adopt the NE mixed strategy after experiencing a tie output is to discourage player Y from choosing action 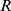: although Y might get a higher expected payoff in one game round by choosing action 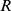 rather than action 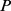, the former choice has a high probability of leading to a tie, which will then reduce player Y's expected payoff to 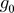 in the following one or even more game rounds.

On the other hand, when 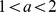, a play output of tie is better off to the group than a win-lose output. Then player X has the option of implementing a CT strategy to discourage player Y from either winning over or losing to her. Again for the simplest case of unit memory length, the recipe of the CT strategy is: If player X ties with player Y in the previous game round, then in the next round she chooses action 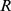 with probability 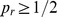 and action 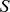 with probability 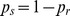; but if X either wins over or loses to Y in the previous round, then in the next round she chooses the three candidate actions with equal probability 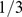.

It turns out that the optimal CT strategy of unit memory length has the following quantitative properties:

1. If 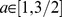, then the optimal value 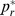 for the choice probability 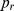 is 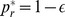, and the optimal EPRs of player X and player Y are equal, 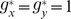.
2. If 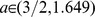, then 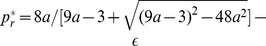, and the optimal EPRs for X and Y are, respectively, 
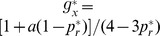
 and 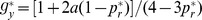.
3. If 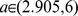, then 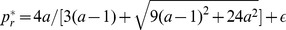, and the optimal EPRs for X and Y are, respectively, 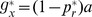 and 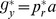.
4. If 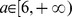, then 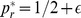, and the optimal EPRs for X and Y are equal, 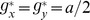.

**Figure 3:**
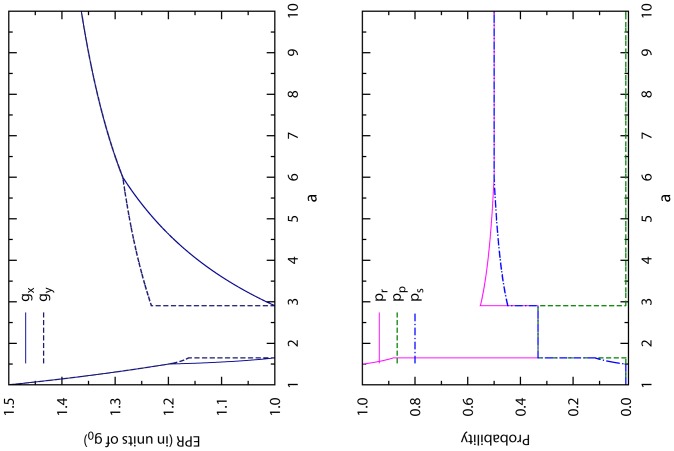
Optimal CT strategy of unit memory length. The optimal values of both players' expected payoff per round 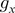 and 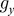 are shown in the upper panel (in units of NE payoff 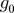) for each fixed value of 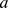, while the optimal values of the CT strategy's choice probabilities 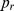, 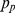 and 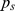 are shown in the lower panel. When 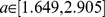 the NE mixed strategy is better for player X than the CT strategy.

Figure 3 gives a direct view of these properties. Compared with the optimal memoryless CT strategy of Fig. 2, we notice a major qualitative improvement is that this new optimal CT strategy achieves fair outcomes to player X and player Y when 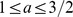 or 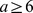. However, this optimal CT strategy of unit memory length is still not perfect, as it is not applicable for 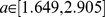, and it is not completely fair to the proactive player X for 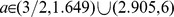.

**Figure 4:**
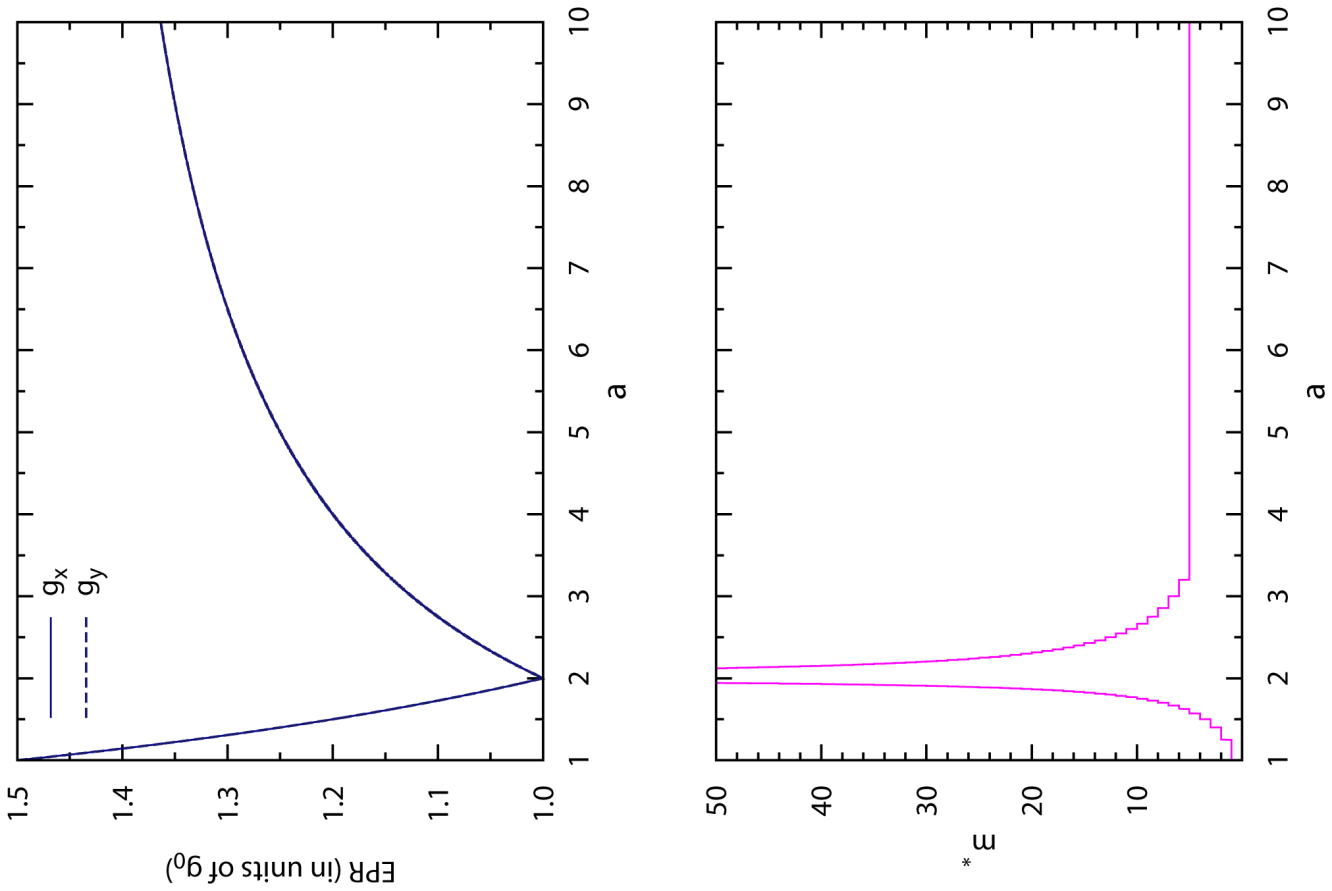
Optimal CT strategy of finite memory length. The optimal values of both players' expected payoff per round 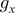 and 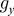 are shown in the upper panel (in units of NE payoff 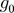) for each fixed value of 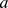, while the minimal memory length 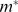 of the CT strategy is shown in the lower panel.

To completely eliminate these undesirable features, player X can increase the memory length of her CT strategy and therefore be more non-tolerant to defection. There are many ways of implementing such an idea. When 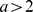, arguably the simplest CT strategy of memory length 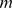 goes as follows: By default player X adopts the mixed strategy 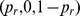 in every game round, namely she chooses action 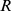 with probability 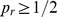 and action 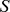 with the remaining probability 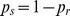; however if a tie occurs in one game round, then player X shifts to the NE mixed strategy 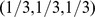 in the next 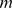 game rounds and then shifts back to the default strategy 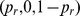 in the (*m*+1)-th game round. It is a simple exercise to check that, if 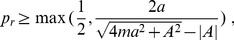
(5)where 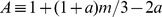, then player Y will be satisfied with sticking to action 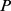 in every game round. If player X sets the memory length to the smallest positive integer 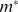 which reduces Eq. (5) to the trivial requirement of 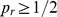, then it is optimal for player X to set 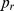 to the value 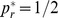, and the optimal EPRs for player X and player Y are equal, 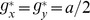. Notice that for 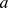 approaches 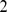 from above with 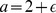, the required minimal memory length diverges as 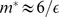. In other words, it is most difficult to enforce fair cooperation when 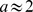, see Fig. 4.

If the payoff parameter 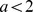, an optimal CT strategy with memory length 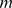 can be constructed following the same line of reasoning as above, namely that player X adopts action 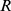 at every game round, but if she loses to player Y in one game round, then she shifts to the NE mixed strategy in the next 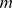 game rounds and then shifts back to the default strategy 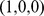 in the 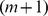-th game round. We can easily verify that if player X sets the memory length to be 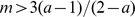, then it is optimal for player Y to stick to action 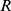 in every game round, and the optimal EPRs for both players are equal, 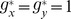.

As clearly demonstrated in Fig. 4, for each payoff parameter 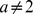, an optimal CT strategy with a finite memory length 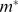 can be implemented to achieve maximal and fair accumulated payoff for both players. At 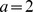, there is no need to adopt a CT strategy, as the NE mixed strategy is itself optimal.

## Discussion

We have demonstrated in this paper that fair cooperation can be achieved in the two-player iterated RPS game. Such a highly cooperative state brings maximal accumulated payoff to the group, and it is not enforced by external authorities but by the proactive decision of one player to adopt an optimal cooperation-trap strategy. The basic designing principle of such optimal CT strategies should be generally applicable to other two-player iterated non-cooperation games.

For the optimal CT strategies to work, the passive player Y is assumed to be considerably rational so that he adopts a best response strategy to that of his opponent X to maximize his accumulated payoff, while the proactive player X is assumed in addition to be wise enough so that she does not exploit the cooperation state of her opponent too much but is satisfied with a fair share of the total accumulated group payoff. This latter assumption might be a little bit too strong, but maybe it is not strictly necessary as player Y will punish X for defection behaviors.

For the iterated RPS game, it appears to be impossible for the proactive player X to design a CT strategy which brings higher expected payoff per game round to herself than to her opponent. However, this is not a general conclusion. For some other game systems, notably the iterated PD game [13], [20]), the proactive player X has the option of optimizing her CT strategy to extort her opponent Y. We do not recommend the adoption of such greedy strategies, as the opponent player Y will very likely be frustrated by the defection behaviors of player X and he may then choose not to cooperate even such a choice hurts also himself [21].

When strategic interactions occur in biological systems [9], [10], the involved individual animals, insects, bacteria, cells,…, are of course far from being rational or sufficiently intelligent. However the collective decision-making of such agents at the population level, aided by the evolutionary mechanism of mutation and selection, may appear to be very rational. By trial and error, such systems may develop certain CT-like strategies even without the need of intelligent designing. It would be very interesting to investigate empirically whether CT strategies are actually implemented in some biological systems, such as the formation of symbiosis relationship between two species.

Cooperation in a finite-population RPS game system with more than two players may be much more difficult to achieve than the case of two players. A recent theoretical investigation by one of the present authors [19] suggested that optimized conditional response strategies might offer higher accumulated payoffs to individual players than the NE mixed strategy does. But it is still an open question as to whether high degree of cooperation can also be enforced in a multiple-player iterated RPS game by a number of proactive players. The case of multiple players interacting through a ring topology might serve as the simplest model system to study. We leave such a challenging issue to future investigations.

The iterated two-player RPS game might also serve as a simple system to quantitatively measure the degree of rationality of single human subjects. For example, an experiment can be arranged as follows. A human subject Y plays repeatedly with a fixed opponent X which is actually a computer implementing an optimal CT strategy. But Y does not know that he is playing with a computer and assumes he is playing with another human subject. By analyzing the evolution trajectory of player Y's action choices, we may quantitative measure the learning behavior of player Y and his tendency of making rational decisions. We are discussing with colleagues about the possibility of carrying out such an experimental study.
